# Multiphysics-Coupled Simulation of Ultrasound-Assisted Tailing Slurry Sedimentation

**DOI:** 10.3390/ma18153430

**Published:** 2025-07-22

**Authors:** Liang Peng, Congcong Zhao

**Affiliations:** 1 School of Resources and Safety Engineering, Central South University, Changsha 410083, China; liangpeng@csu.edu.cn; 2 State Key Laboratory of Safety Technology of Metal Mines, Changsha Institute of Mining Research Co., Ltd., Changsha 410012, China; 3 School of Resources and Safety Engineering, University of Science and Technology Beijing, Beijing 100083, China

**Keywords:** tailing slurry, thickening sedimentation, ultrasonic radiation, sound pressure distribution, multiphysics coupling

## Abstract

This study establishes a multiphysics coupling model of acoustics, mechanics, and electrostatics through COMSOL, systematically explores the sound field distribution and stress–strain characteristics of tailing particles in sand silos under different frequencies of ultrasonic radiation, and proposes an optimization scheme for the sound field. The simulation results show that under 28 kHz ultrasonic radiation, the amplitude of sound pressure in the sand silo (173 Pa) is much lower than that at 40 kHz (1220 Pa), which can avoid damaging the original settlement mode of the tail mortar. At the same time, the periodic fluctuation amplitude of its longitudinal sound pressure is significantly greater than 25 kHz, which can promote settlement by enhancing particle tensile and compressive stress, achieving the best comprehensive effect. The staggered placement scheme of the transducer eliminates upward disturbance in the flow field by changing the longitudinal opposing sound field to oblique propagation, reduces energy dissipation, and increases the highest sound pressure level in the compartment to 130 dB. The sound pressure distribution density is significantly improved, further enhancing the settling effect. This study clarifies the correlation mechanism between ultrasound parameters and tailings’ settling efficiency, providing a theoretical basis for parameter optimization of ultrasound-assisted tailing treatment technology. Its results have important application value in the optimization of tailings settling in metal mine tailing filling.

## 1. Introduction

The outline of China’s 14th Five Year Plan proposes two important goals of “ensuring energy security and promoting green transformation and development”, among which green mining is the primary method to achieve green transformation and development, as well as energy security [1,2]. As one of the three major mining methods, backfill mining can effectively control surface subsidence, reduce underground fine mud pollution, and minimize hazardous waste emissions, gradually becoming the primary choice for mining construction [3,4]. The filling mining method mainly uses paste filling and includes three major processes—solid–liquid separation, mixing, and transportation. The paste thickener, as an important equipment for solid–liquid separation of tailing slurry, is widely accepted by mines due to its advantages such as large capacity and high efficiency [5]. With the decrease in mineral resource grade and the reform and upgrading of beneficiation technology, the particle size of tailings is becoming finer and finer [6,7]. In the 1950s, the proportion of tailings with a size of 200 mesh was 60–70%. Nowadays, most mines have a proportion of over 90%, and some even reach over 95%. To cope with this situation, methods such as increasing the height of the mortar layer, adding sufficient flocculants, or prolonged concentrated settling time are often used. However, the high filling cost, cumbersome thickening process, and unstable concentration of tail mortar caused by this have become important factors restricting the development of paste filling. Therefore, it is urgent to propose new methods and equipment to accelerate the thickening and settlement of tail sand [8].

Due to its continuous maturity, ultrasound technology has shone in various fields. As a special energy output method, researchers at home and abroad have attempted to promote ultrasound to enhance particle aggregation and sedimentation [9,10,11,12,13]. Vera Vikulina et al. conducted a study on the effect of ultrasonic vibration on the solid coagulation process of suspensions, and established a mathematical relationship between the amount of flocculant added, the duration of ultrasonic action, and the coagulation settling effect. Increasing the duration of ultrasonic action is beneficial for reducing the amount of flocculant added. Wang Jinlin [14]. explored the effects of different parameters of low-intensity ultrasound at low temperatures on effluent quality, sludge characteristics, and microbial community structure. The optimal ultrasound sound energy density, action time, and interval time were established, and the mechanism of ultrasound enhancement on sewage was revealed by coupling a single action with continuous operation of SBR. In terms of settling of tailing particles in mines, many research institutions have conducted relevant studies [15,16]. Jin rui [17] conducted research on the motion characteristics of different graded minerals on inclined plate surfaces of various materials, focusing on the difficulties in feeding and surface consolidation of inclined plate thickeners. The results showed that applying a certain amount of excitation force on the inclined plate surface is beneficial for particle grading and, to some extent, prevents surface consolidation. Jiho Park [18] et al. investigated the effects of acoustic power, duration, and initial moisture content on the self-weight consolidation of clay minerals. They found that under the action of ultrasound, the time required for the same settlement amount was shorter, the consolidation time was significantly shortened, and the final settlement rate of the slurry was greatly increased. Svetlana Samchenko [19] et al. conducted relevant settling tests on refractory finely ground slag (FGS) and established the optimal parameters for ultrasonic dispersion of slag suspension. They found that compared with ordinary mixed settling, the aggregation and settling stability of FGS suspension were improved by 23 times under the action of ultrasonic waves. Jianhua Du et al. [20] conducted a study on the variation law of the settling layer of tail mortar under ultrasonic treatment. After ultrasonic treatment, the settling layer became denser, and the density increased from 33.72 wt.% to 37.48 wt.%. The above research indicates that ultrasound has a positive effect on particle settling, and moderate ultrasound action has a good effect on the thickening effect of tailings. However, most studies use post-processing methods to analyze the settlement effect data by changing the dense conditions, lacking the display of the internal acoustic field characteristics of the equipment under ultrasonic radiation, as well as the stress–strain situation of tailing particles in the acoustic field.

Generally speaking, the thickening and settling of tailings mainly rely on the gravity of tailing flocs supplemented by mechanical structures such as rakes for forced dewatering [21,22]. However, with the promotion and application of high-efficiency thickeners, problems such as large fluctuations in tailing concentration, inability to break through the processing capacity of thickeners, and pressure on rakes have emerged [22,23,24]. To this end, a new treatment method has been introduced, which is to apply ultrasonic waves with a wide radiation range and strong penetration ability to the settling stage of tailings [25]. Ultrasonic frequency is a commonly used process parameter in engineering, corresponding to the number of ultrasonic actions per unit time. Research mainly focuses on the effect of different ultrasonic frequencies on tailing settling. The ultrasonic effect on tailing settlement mainly transfers the energy generated by high-speed vibration to the tailings in the variable concentration zone and compression consolidation zone through oscillators and other media, and forces the stationary tailing flocs to move again through mechanical and cavitation effects [26,27], promoting the reformation of water channels and forcing the tailings in the variable concentration zone and compression consolidation zone to undergo “secondary dehydration” [28,29].

In the engineering application of ultrasonic-guidance-assisted tail mortar thickening, there are currently problems such as unclear sound field parameters and particle settling response laws, and an unclear influence mechanism of transducer layout on thickening efficiency. This situation seriously restricts the efficient application of ultrasonic technology in metal mine tail sand thickening equipment. Based on this, the core purpose of this study is to reveal the sound pressure distribution characteristics inside the thickening equipment under ultrasonic guidance and the stress–strain laws of settling particles, clarify the inherent mechanism of ultrasonic acceleration of tail mortar thickening, and provide theoretical support for optimizing the ultrasound-assisted thickening process in the field of metal mine filling. Specific research objectives include using COMSOL 6.0 to build a multi-field-coupled simulation model of sound field, stress field, and electrostatic field using software to quantitatively analyze the spatial distribution characteristics of the sound field inside the thickening equipment and the variation law of the stress–strain of tailing particles. Based on the acoustic field characteristics generated by the transducer, a targeted optimization plan for the installation position of the transducer is proposed to enhance the promoting effect of ultrasonic waves on the thickening of tailings mortar, and provide technical reference for efficient thickening and safe filling of tailings in metal mines.

## 2. Experimental Overview

### 2.1. Tailing Material

**Figure 1:**
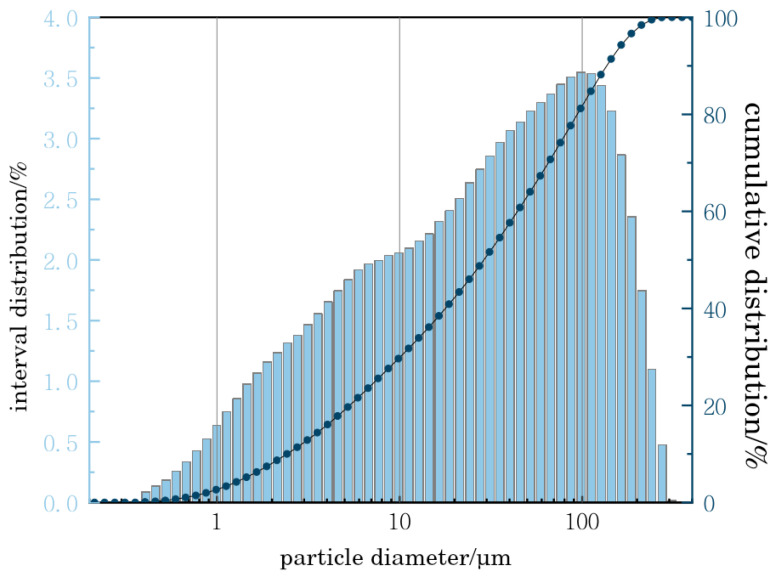
Composition of tail sand particle size.

The tailing aggregate required for the experiment comes from a lead–zinc mine in Jiangxi, with particle size composition shown below. The proportion of tailing particles with a particle size of less than 74.000 μm (−200 mesh) in the tailing sample is about 74%, and the proportion of particles with a particle size ranging from 0.000 μm to 31.100 μm is about 50%. According to the measured particle size distribution (Figure 1), these tailings belong to fine tailings.

The following are the equivalent particle sizes of tailings: d10 = 1.15, d50 = 19.96, d90 = 142.66. Spectral analysis was used to analyze the chemical composition of all tailings. The experimental results showed that the main components of all tailings were SiO_2_, CaO, and Al_2_O_3_, and the recoverable metal content was relatively low. These did not contain or contained very little toxic and harmful substances, which met the environmental protection requirements for underground filling and could be used as filling aggregates.

### 2.2. Research Plan

To investigate the characteristics of sound field changes and tailing particle transport in the thickener at different frequencies, three different ultrasonic frequencies were simulated, as shown in Table 1. The simulation model is made of iron, with a height of 540 mm and a cross-section of 160 mm × 160 mm square. The selection of the ultrasonic frequency range (25–40 kHz) in this article is based on the experimental results of previous scholars [30] and the team’s previous related research.

## 3. Construction of Numerical Models

**Figure 2:**
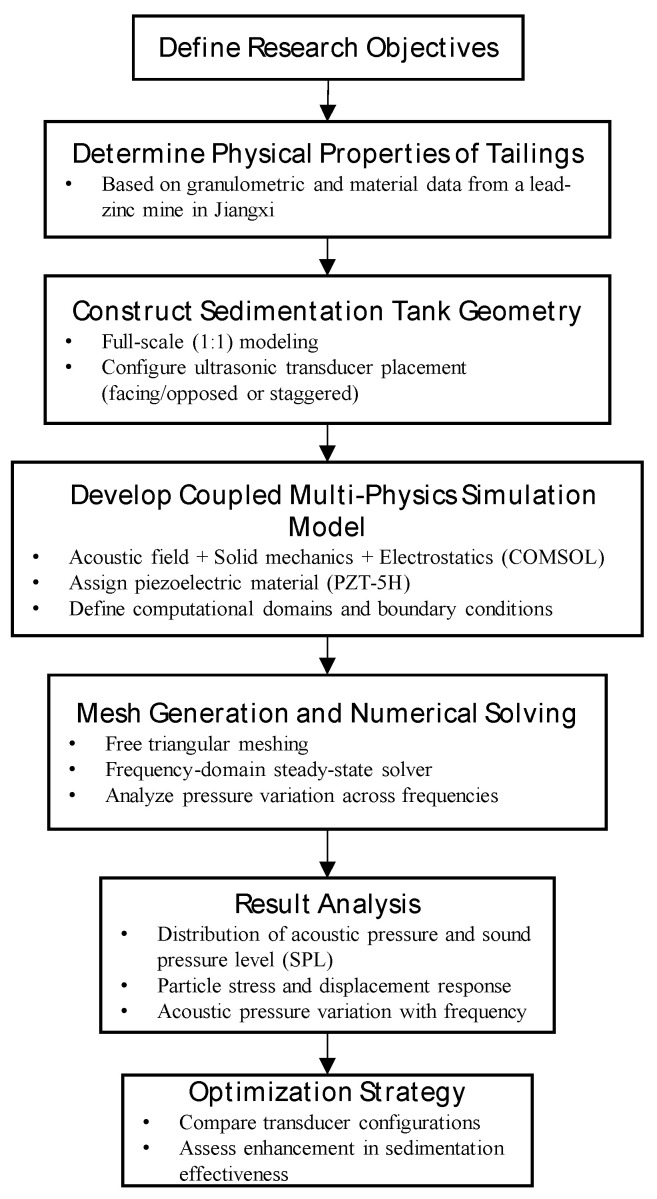
Experimental research flowchart.

The application of ultrasound in the thickening and settling process of tailings involves multiphysics fields such as mechanics and acoustics. To ensure the reliability of the results, the simulation adopts a multi-field-coupling method. With the help of COMSOL 6.0 multi-field-coupling numerical simulation software, the characteristics of sound field changes inside the thickener and the migration law of tailings under different ultrasound frequencies are studied (Figure 2).

### 3.1. Mathematical Model

The calculation uses a steady-state frequency-domain solver, and the frequency-domain study simulates wave propagation using equations from linear fluid dynamics (pressure waves) and structural dynamics (elastic waves). The linearized isentropic state equation is adopted, assuming lossless and adiabatic flow while ignoring viscous effects. Under these assumptions, the sound field is described by a variable, namely, pressure (unit: Pa), and controlled by the wave equation:

(1) $$\nabla \left\{-\frac{1}{\rho_{c}}\left(\nabla \rho_{t}-q_{d}\right)\right\}-\frac{\omega^{2}p}{\rho_{c}c^{2}}=0$$

(2) $$p=p_{0}e^{iw\cdot t}$$

(3) $$\nabla \left\{-\frac{1}{\rho_{c}}\left(\nabla \rho_{t}-q_{d}\right)\right\}-\frac{k_{eq}^{2}p_{t}}{\rho_{c}}=2\sqrt{\frac{p\omega }{p_{c}}}$$

(4) $$k_{eq}^{2}={\left(\frac{\omega }{c}\right)}^{2}$$

(5) $$c_{c}=\frac{\omega }{k},k=\frac{\omega }{c}-i\alpha ,p_{t}=p+p_{b}$$

In the formula,

– $\rho_{c}$—medium density, kg/m^3^;
– *q*—Dipole source, N/m^3^;
– ω —Angular frequency, rad/s;
– c—Speed of sound, m/s;
– t—Time, s;
– $p_{t}$—The sound pressure changes at different times, Pa;
– $k_{eq}$—Equivalent wave velocity.

The wave equation of ultrasound propagation in a medium is as follows:

(6) $$c^{2}\frac{\partial^{2}u}{\partial x^{2}}+2c\alpha v\frac{\partial u}{\partial x}=\frac{\partial^{2}u}{\partial t^{2}}$$

t—time, s.

The coupling boundary adopts a weak expression:

“(-treatasconst(acpr.nx) ×asb1.u_ttx − treatasconst(acpr.ny) × asb1.u_tty − treatasconst (acpr.nz) × asb1.u_ttz) × test(p) × acpr.delta”

The variation in acceleration on the boundary is described, where asb1. u_ttx, asb1. u_tty, and asb1. u_ttz represent the acceleration of the structure in the x, y, and z directions, acpr.nx, acpr.ny, and acpr.nz represent the unit normal phasor components of the boundary, test (p) represents the test function for pressure, and acpr.deta is the scaling factor.

“acpr.FAcoPerAreax × test(asb1.ux) + acpr.FAcoPerAreay × test(asb1.uy) + acpr.FAcoPerAreaz × test(asb1.uz)”

The description regards the effect of sound radiation force on the materials inside the warehouse, where acpr.FAcoPerAreax, acpr.FAcoPerAreay, and acpr.FAcoPerAreaxz are the sound radiation forces per unit area in the x, y, and z directions, respectively.

By using the treatasconst command, the sound pressure in the x, y, and z directions and the vibration velocity in the directions are converted into each other. acpr.FAcoPerAreax, acpr.FAcoPerAreay, and acpr.FAcoPerAreaxz are the sound radiation forces per unit area in the x, y, and z directions, respectively. The ultrasonic transducer transmits the vibration to the sand bin through the coupling boundary, and the sound propagates in the mortar. After encountering particles, the sound energy is converted into sound radiation force through the coupling boundary to promote particle movement and vibration.

### 3.2. Sand Silo Model

**Figure 3:**
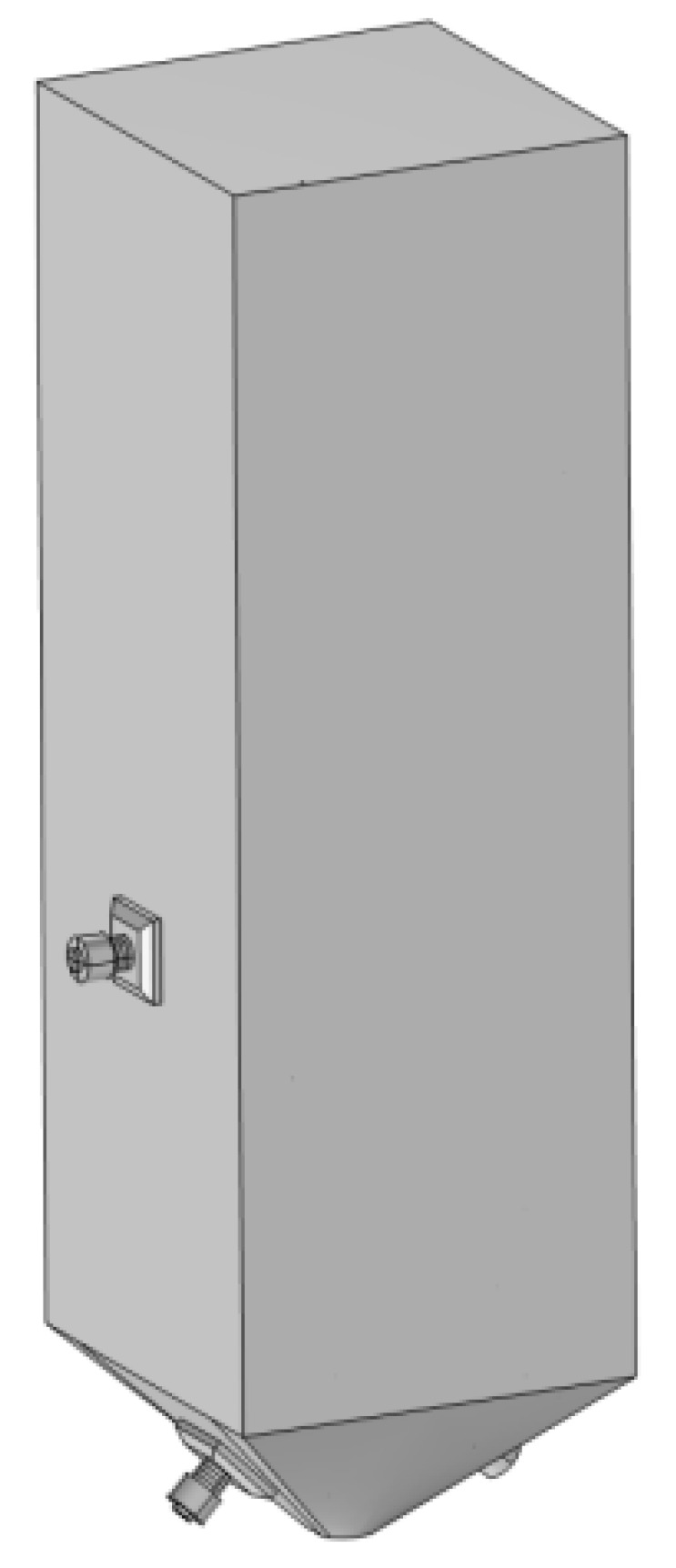
Sand silo model.

The structure of the sand silo model adopts a 1:1 ratio to the actual size, with a height of 540 mm and a cross-section of 160 mm × 160 mm square. The bottom adopts a conical surface with a cone angle of 45°. Two ultrasonic transducers on opposite sides are placed on both sides of the model and at the bottom cone angle. The transducer size is 50 mm × 30 mm, and the distance between the transducer center and the bottom is 150 mm. The model is shown in Figure 3.

#### 3.2.1. Condition Setting

**Figure 4:**
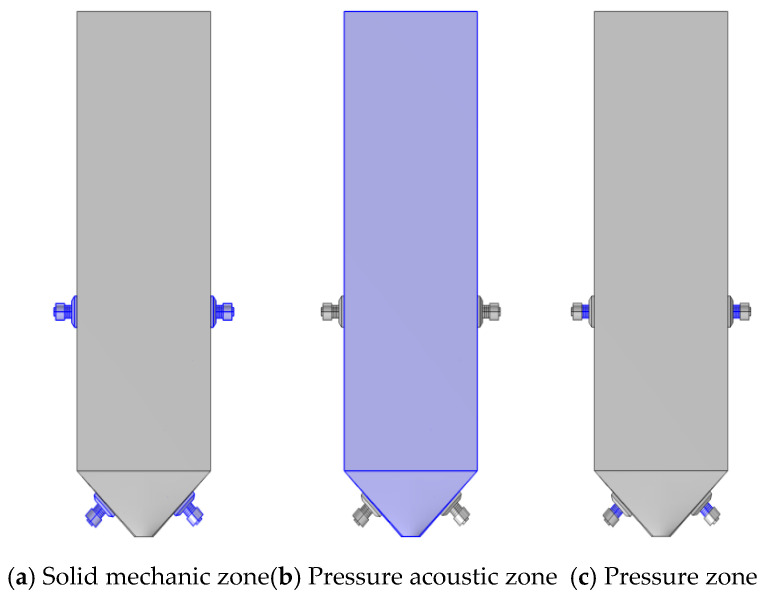
Regional division.

The calculation is carried out by coupling three physical fields: the pressure acoustic frequency domain module, the solid mechanic module, and the electrostatic module, as shown in Figure 4, which are divided into three regions: the solid mechanic region of the ultrasonic transducer; the pressure acoustic zone inside the model sand silo; and the pressure zone where the ultrasonic transducer comes into contact with the chamber wall.

- Piezoelectric materials

The coupling structure is the piezoelectric effect, which refers to the deformation of piezoelectric materials when subjected to an external electric field, and vice versa. In a coupled structure, sound waves need to be able to be transmitted from the piezoelectric material to the structural material in order to achieve effective energy conversion. The piezoelectric material parameters selected for simulation are PZT-5H type piezoelectric material, with material properties shown in Table 2. This widely used lead zirconate titanate ferroelectric ceramic has good piezoelectric properties. When an electric field is applied, the piezoelectric ceramic will deform, causing the generation or propagation of sound waves.

- Tail mortar

The tail mortar area is the pressure acoustic zone in Figure 4, with environmental conditions of a standard atmospheric pressure, temperature of 20 °C, and sound velocity of 1500 m/s. The material properties of the tail mortar slurry are shown in Table 3.

#### 3.2.2. Grid Division

**Figure 5:**
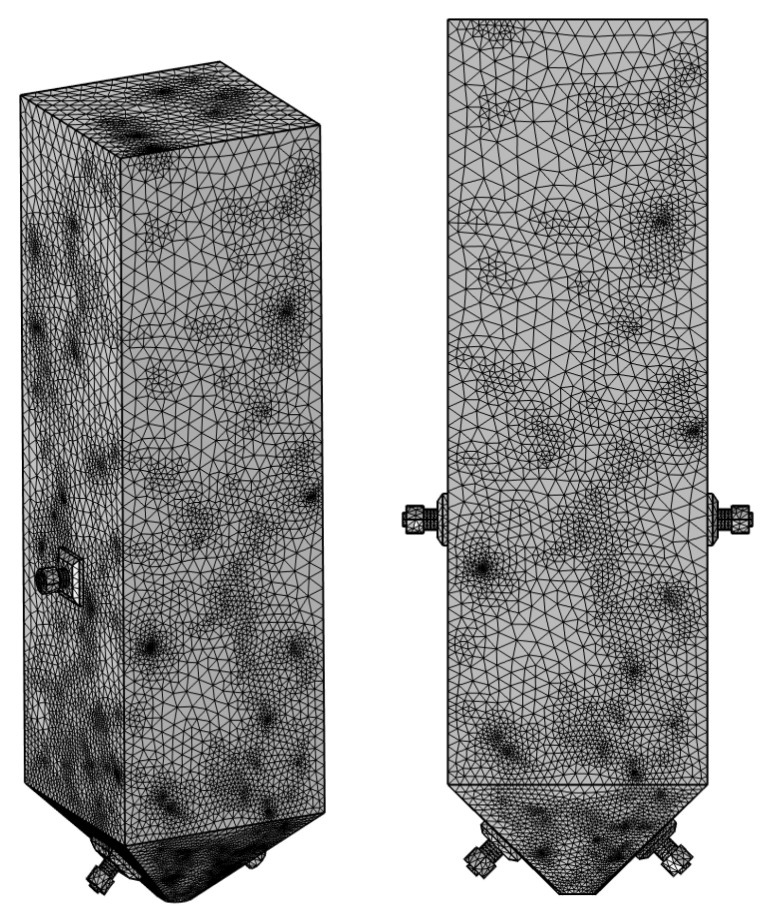
Schematic diagram of grid division.

The geometric mesh division is shown in Figure 5, which adopts a free mesh triangular mesh division. The simulation involves three frequencies of 25 kHz, 28 kHz, and 40 kHz, and the higher the frequency, the shorter the wavelength. The wavelength of 40 kHz ultrasound in liquid is 3.8 cm, and in solid, it is 5.0 cm. During simulation, at least 4 to 6 meshes should be included in one wavelength, resulting in a total of 6,121,861 meshes.

## 4. Results’ Analysis

The transducer has a power of 100 W and can deliver 100 joules of energy per second to the coupling structure. The calculated frequencies are 25 KHz, 28 KHz, and 40 KHz. In order to reduce computational complexity, the number of particles to be added is 2200, and they are added based on the proportion of particle size. The main focus is on studying the sound radiation force received by particles under the action of sound field. The common settlement experiment involves too many particles, and the number of computational grids can reach tens of billions or even more, making it impossible to perform finite element simulation calculations.

### 4.1. Analysis of Sound Intensity Results

#### 4.1.1. Sound Pressure

**Figure 6:**
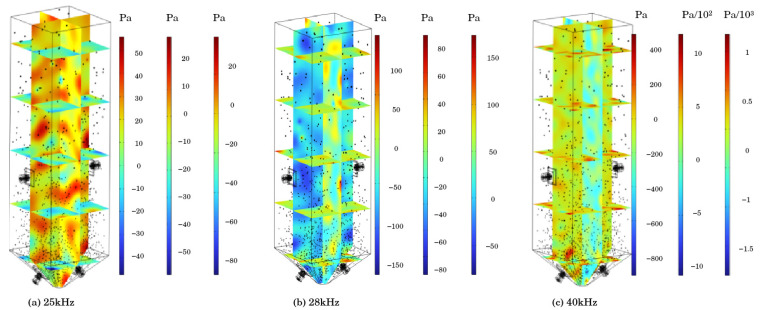
Sound pressure cloud map.

As shown in Figure 6, the cloud maps of the total sound pressure inside the sand silo under the action of ultrasonic frequencies of 25 kHz, 28 kHz, and 40 kHz are taken. Five cross-sections in the *Z*-axis direction and the cross-section of the center surface of the sand silo in the *X-* and *Y*-axis directions are taken. From the graph, it can be observed that the highest sound pressure value in the sand bin reached 55.9 Pa under the action of ultrasonic waves with a frequency of 25 kHz, 173 Pa under the action of ultrasonic waves with a frequency of 28 kHz, and 1220 Pa at 40 kHz. Under the same power input, the higher the frequency of the sound waves, the greater the energy generated. High-frequency sound waves have a shorter wavelength, and energy is concentrated in a smaller spatial range. The energy distribution at the same power is denser, and the energy density per unit area increases. This means that high-frequency sound waves carry more energy per unit area and exert greater pressure per unit area, resulting in higher sound pressure levels.

#### 4.1.2. Sound Pressure Level

**Figure 7:**
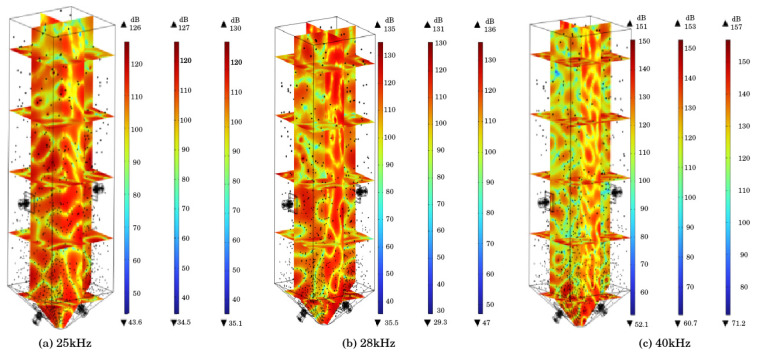
Acoustic field distribution.

The sound field distribution of the mortar domain is shown in Figure 7, which is the cloud map of the total sound pressure level inside the sand silo under the action of ultrasonic frequencies of 25 kHz, 28 kHz, and 40 kHz. Five cross-sections in the *Z*-axis direction and the center plane cross-section of the sand silo in the *X-* and *Y*-axis directions were taken. From Figure 6, it can be observed that as the frequency increases, the sound pressure level inside the silo gradually increases. When the ultrasonic frequency is 25 kHz, the highest sound pressure level inside the silo is 130 dB; at 28 kHz, the highest sound pressure level inside the silo reaches 136 dB; and at 40 kHz, the highest sound pressure level inside the silo reaches 157 dB.

**Figure 8:**
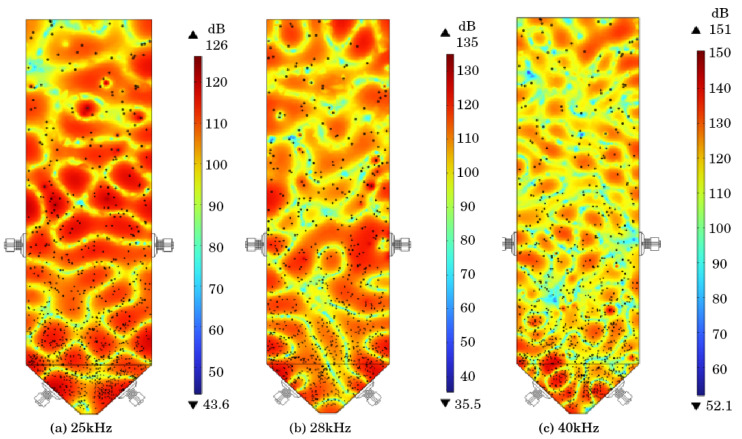
Sound pressure level cloud map.

Extracting the *Y*-axis cross-section from the sound pressure level cloud map of the sand silo, as shown in Figure 8, it can be observed that as the ultrasonic frequency increases, the change in sound pressure level gradient per unit area increases. The energy density of high-frequency sound waves is higher, and the tensile and compressive stresses formed by cavitation on a unit area are greater, resulting in higher sound pressure levels and greater disturbance to the tailing slurry inside the sand silo. In addition, compared to the distribution of sound pressure levels in the horizontal direction, as the frequency increases, the effect of sound waves propagating horizontally becomes stronger, and the directionality of the ultrasonic transducer improves.

### 4.2. Analysis of Particle Force Results

**Figure 9:**
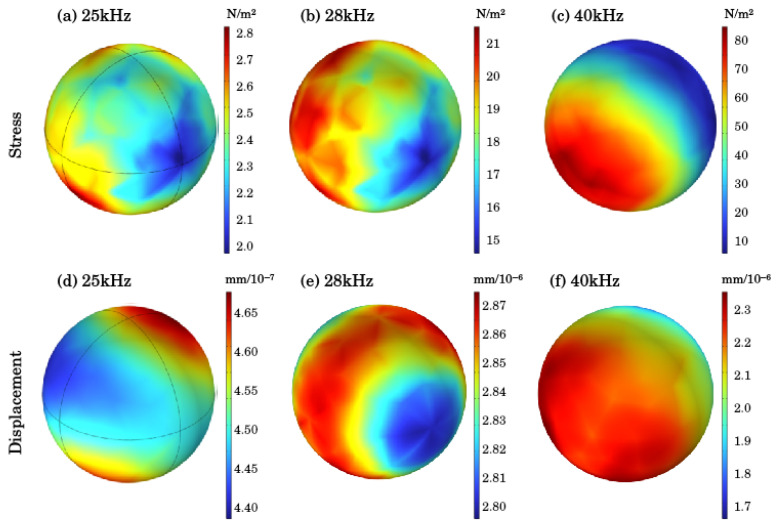
Stress and displacement of tailing particles. (**a**) Stress at 25 kHz, (**b**) Stress at 28 kHz, (**c**) Stress at 40 kHz, (**d**) Displacement at 25 kHz, (**e**) Displacement at 28 kHz, (**f**) Displacement at 40 kHz.

In order to explore the influence of ultrasonic irradiation on tailing particles, based on the investigation of the changes in sound pressure and sound pressure level inside the silo under the action of different frequencies of ultrasonic waves, the tailing particles in the sand silo were analyzed, as shown in Figure 9, which shows the stress and displacement of tailing particles at the same position inside the sand silo. (a), (b), and (c) are stress cloud maps, and (d), (e), and (f) are displacement cloud maps. From Figure 8, it can be observed that as the ultrasonic frequency increases, the stress and displacement of tailing particles gradually increase. When the ultrasonic frequency is 25 kHz, the stress on tailing particles is 2.81 N/m^2^ and the displacement is 4.69 × 10^−7^ mm. However, when the frequency increases to 28 kHz, the stress on tailing particles is 21.8 N/m^2^ and the displacement is 2.88 × 10^−6^ mm. At 40 kHz, the stress reaches 83.9 N/m^2^ and the displacement is 2.31 × 10^−6^ mm.

### 4.3. Comparison of Sound Intensity Characteristics: Taking Sound Pressure Collection as an Example

**Figure 10:**
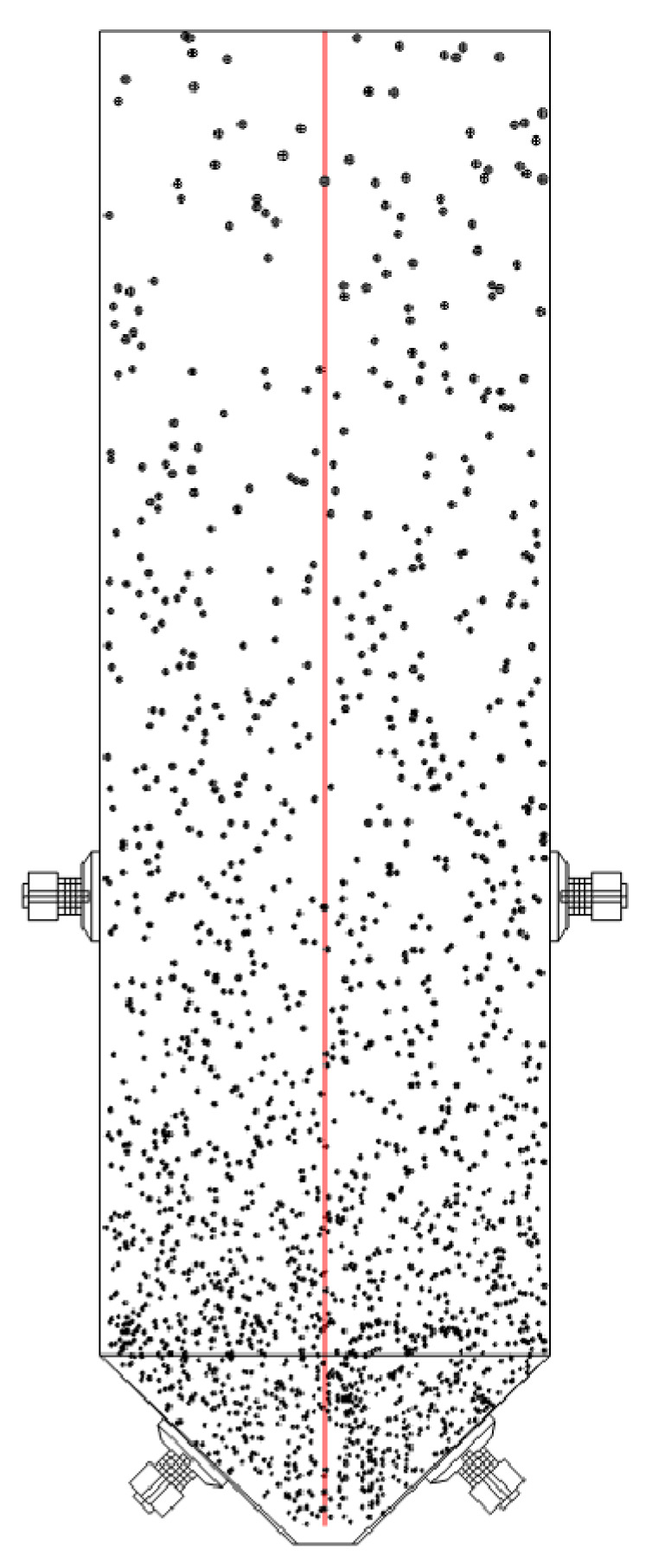
Position of sound-pressure-sampling axis.

The sound pressure is sampled at the center of the sand silo, the sound pressure level is collected, and the distribution of sound pressure under the action of ultrasonic waves is quantitatively analyzed. The sampling method is shown in Figure 10. A sampling axis is set up for sampling, with the center point of the top of the model as the origin and the center point of the cone bottom as the endpoint.

**Figure 11:**
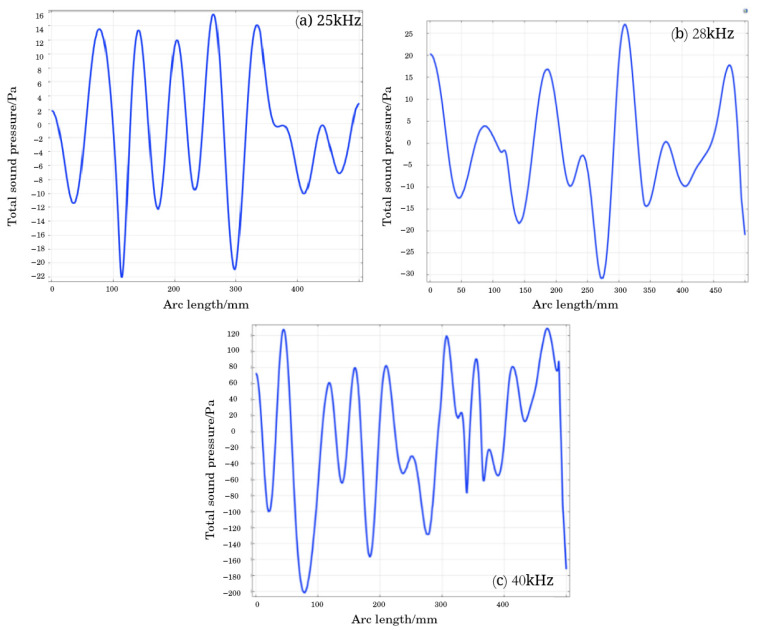
Sampling sound pressure curve graph.

As shown in Figure 11, the sound pressure curve measured on the sampling axis is shown. It can be observed from the figure that as the ultrasonic frequency increases, the amplitude of the sound pressure curve increases, and the total sound pressure amplitude fluctuation value increases from ±22 Pa at 25 kHz to ±30 Pa at 28 kHz, with the maximum amplitude at 40 kHz, which is ±200 Pa. Due to the microstructure and characteristics of solid particles in the tail mortar, as well as the interaction between sound waves and materials, high-frequency ultrasonic waves propagate in the tail mortar, which are affected by obstacles at the microscopic scale and the non-uniformity of the fluid medium, resulting in the obstruction of the propagation path, scattering phenomena, and energy loss, leading to spatial fluctuations in sound pressure values. At 25 kHz and 28 kHz, the amplitude of sound pressure values on the axis shows a trend of 0 Pa. At 40 kHz, the amplitude range is positively correlated with the arc length and gradually decreases as the centerline shrinks.

Using an arc length of 0 mm as the origin and dividing the sound pressure curve into two highest points and one lowest point as a cycle, it can be observed that in (a), the amplitude changes of adjacent cycles on the sound pressure curve are small, and the tensile and compressive stresses on the tailing particles between cycles are small, resulting in small Brownian motion of the particles. In (c), when the amplitude of the stress curve exceeds 300 Pa, the tailing particles are subjected to high stress, which is not conducive to the settling of the tailing particles. However, under the action of 28 kHz ultrasound, the sound pressure difference between adjacent period amplitudes on the stress curve is large, and the tailing particles are subjected to high stress, with obvious Brownian motion, and the sound pressure amplitude is around 30 Pa, which does not damage the original coagulation settling mode of the tailing particles in the sand silo.

### 4.4. Effect Improvement: Transducer Position Transformation

**Figure 12:**
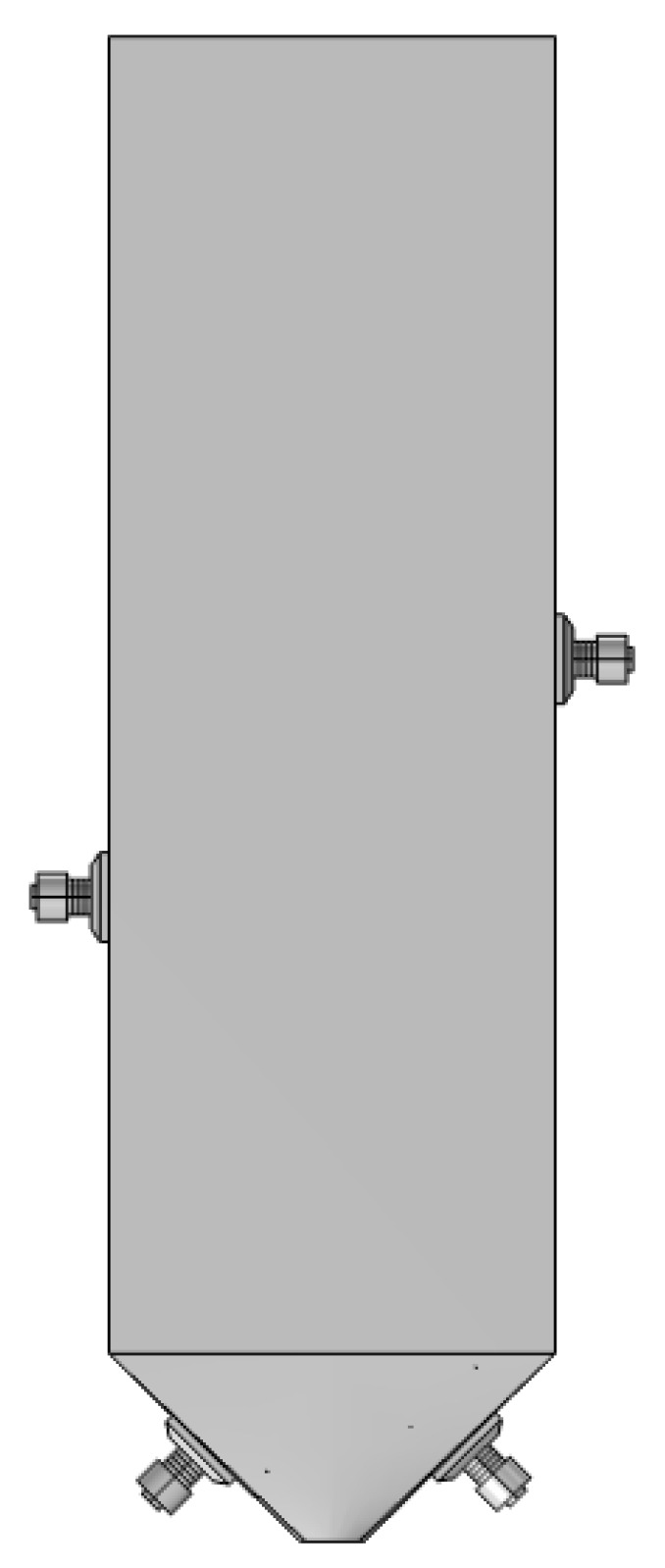
Optimization of transducer position.

**Figure 13:**
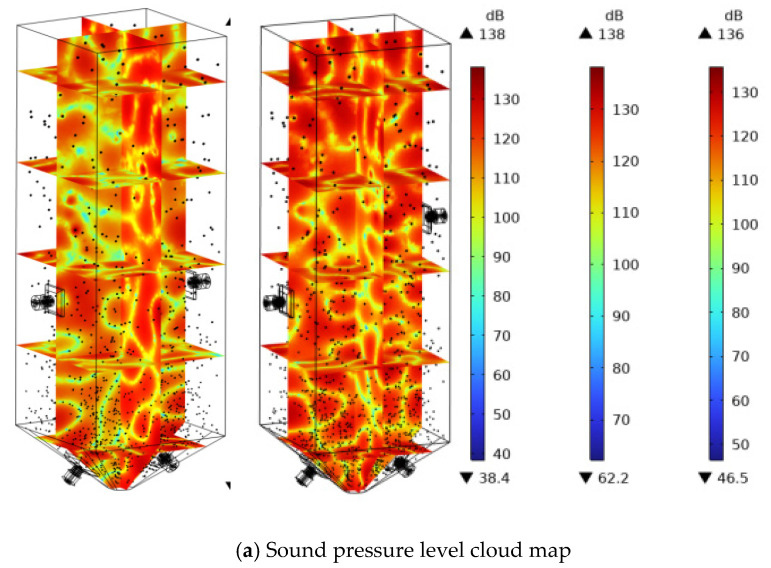

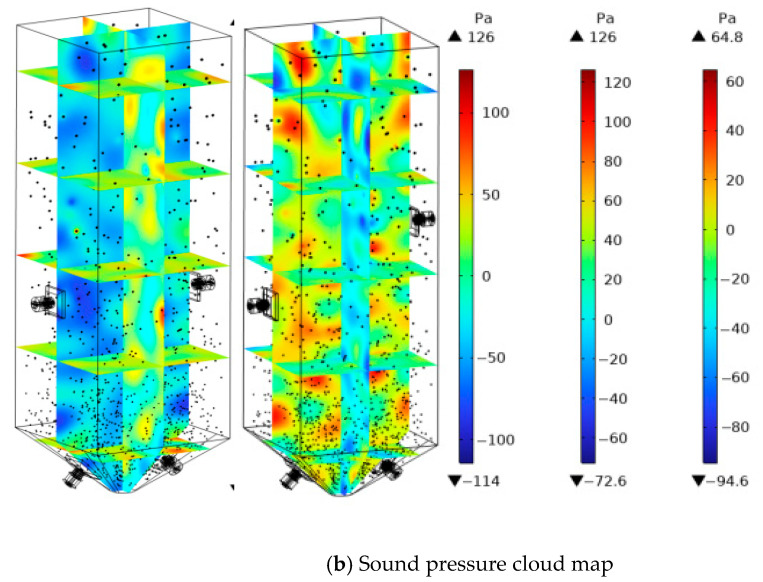
Sound pressure level and cloud map inside the sand silo before and after optimization.

When considering the placement of transducers in opposite positions, there is a phenomenon of opposite impact on the sound field formed inside the sand silo. When two sound fields come into contact, an upward sound wave field will be formed, which will affect the settling process of tailings. Therefore, it is proposed to optimize the placement position of the transducer, with a sound wave frequency of 28 kHz, as shown in Figure 12, and change it to a staggered placement. The sound pressure level and sound pressure cloud map inside the sand silo before and after optimization are shown in Figure 13.

**Figure 14:**
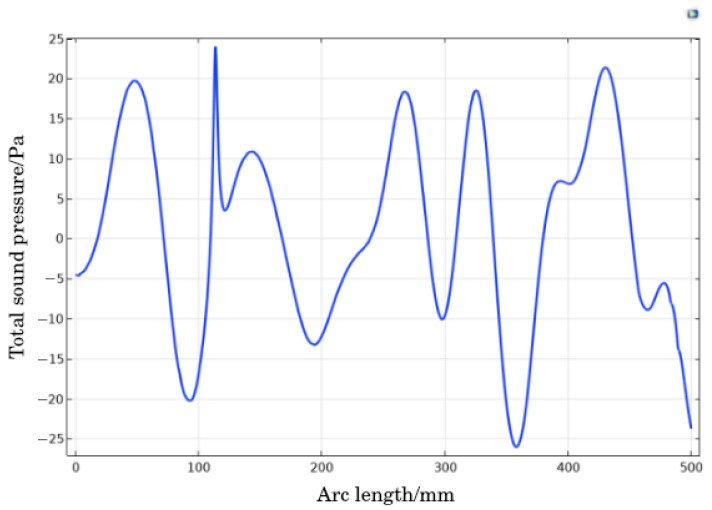
Sound pressure fluctuation curve at the axis position.

By comparing the sound field cloud maps before and after optimization, it was found that the total sound pressure level of the sound field under misaligned placement was mainly between 115 and 130 dB, while the total sound pressure level of the sound field before optimization was slightly lower, mainly between 95 and 125 dB. After optimization, the sound pressure inside the sand silo was also improved to a certain extent. In addition, the staggered placement changes the counteracting phenomenon of resonant ultrasonic waves inside the sand silo when placed opposite to each other in an oblique direction, avoiding an upward sound wave field and greatly reducing the loss of ultrasonic irradiation energy. It increases the sound pressure level and the distribution density of sound pressure inside the silo. The sound pressure values on the axis are recorded and the arc length sound pressure value curve is plotted, as shown in Figure 14. The amplitude is equivalent to the sound pressure amplitude before optimization, and the amplitude changes between adjacent periods are large, which means that the tailing particles in this acoustic field are subjected to greater longitudinal tensile and compressive stress, and the settlement changes are more obvious. That is, compared to the transducer opposed placement mode, staggered placement is more conducive to the precipitation of tailing particles.

## 5. Conclusions

(1) Under the same power input, the energy density per unit area is positively correlated with the sound pressure level. In the scenario of dense tailings in mines, this energy concentration directly affects the strength of the effect on tailing particles. Specific data shows that when the frequency is increased from 25 KHz to 40 KHz, the maximum sound pressure level in the sand silo increases from 130 dB to 157 dB. High-frequency sound waves can generate stronger energy impact on the tailings in local areas of the sand silo, which can quickly act on the locally accumulated tailing particles.
(2) In the sand silo filled with dense tailings in the mine, the longitudinal sound pressure distribution of different frequency sound waves shows periodic fluctuations, but the effect on tailings settlement varies significantly. The maximum sound pressure of a 40 kHz sound wave reaches 1220 Pa. In tailing thickening operations, excessively high sound pressure can excessively disturb the settling tailing particles, which is not conducive to rapid thickening of tailings. Compared with 25 kHz, although the amplitude increase in sound pressure is limited at 28 kHz, the periodic fluctuation in longitudinal sound pressure is more severe, which significantly increases the tensile and compressive stress borne by tailing particles during the settling process. This can effectively promote particle aggregation and settling, which is more in line with the requirements of rapid and efficient settling in mining tailing filling and thickening projects and has better engineering applicability.
(3) In the process of filling tailings and thickening operations in mines, the technical adjustment of changing the longitudinal opposition of resonant sound waves to oblique propagation has brought significant positive effects. It not only eliminates the interference of upward sound field and avoids the tail sand particles being lifted upwards and unable to settle smoothly, but it also reduces energy loss so that the sound pressure level and sound pressure distribution density in the sand silo are synchronously increased. From the perspective of optimizing sound field characteristics, this directly enhances the settling driving effect on tailing particles, allowing for faster and more uniform settling of tailings in the sand silo. It is a key technological improvement to improve the settling efficiency during the process of filling tailings in mines.

This study reveals the dynamic dependence of stress distribution, particle migration, and sedimentation effects during the settling process of tail slurry through multiphysics field modeling of coupled particle–fluid interactions. In addition, the innovative application of ultrasonic methods not only expands the applicable field of high-concentration slurry settlement monitoring but also verifies the correlation mode between solid concentration gradient and acoustic response in theoretical models. These findings provide new scientific understanding of the settlement mechanism of complex slurries and lay the foundation for optimizing industrial monitoring technology.

## Figures and Tables

**Table 1:** Simulation scheme.

Group	G1	G2	G3
Power/W	100	100	100
Frequency	25 kHz	28 kHz	40 kHz

**Table 2:** PZT-5H piezoelectric material properties PZT-5H piezoelectric material properties.

Attribute	Name	Value	Unit
Density	Rho	7500	kg/m^3^
Elastic matrix	cE	{1.27205 × 10^11^, 8.02122 × 10^10^, 1.27205 × 10^11^, 0, 0, 0, 2.29885 × 10^10^, 0, 0, 0, 0, 2.29885 × 10^10^, 0, 0, 0, 0, 0, 2.34742 × 10^10^}	Pa
Coupling matrix	eEs	{0, 0, −6.62281, 0, 0, −6.62281, 0, 0, 23.2403, 0, 17.0345, 0, 17.0345, 0, 0, 0, 0, 0}	C/m^2^
Relative dielectric constant	Eps	{1704.4, 1704.4, 1433.6}	1
Heat capacity at constant pressure	Cp	440	J/(kg·K)
Thermal conductivity	k	1.3	W/(m·K)

**Table 3:** Material properties of tail mortar.

Attribute	Name	Value	Unit
Density	Rho	1800	Kg/m^3^
Dynamic viscosity	mu	2	Pa·s
Specific heat rate	ga	1	1
Conductivity	sig	5.5 × 10^−6^	S/m
Thermal conductivity	k	K(T[1/K])	W/(m·K)

## Data Availability

The original contributions presented in this study are included in the article. Further inquiries can be directed to the corresponding author.
